# SEQADAPT: an adaptable system for the tracking, storage and analysis of high throughput sequencing experiments

**DOI:** 10.1186/1471-2105-11-377

**Published:** 2010-07-14

**Authors:** David B Burdick, Chris C Cavnor, Jeremy Handcock, Sarah Killcoyne, Jake Lin, Bruz Marzolf, Stephen A Ramsey, Hector Rovira, Ryan Bressler, Ilya Shmulevich, John Boyle

**Affiliations:** 1 Institute for Systems Biology, 1441 North 34th Street, Seattle, WA 98103, USA

## Abstract

**Background:**

High throughput sequencing has become an increasingly important tool for biological research. However, the existing software systems for managing and processing these data have not provided the flexible infrastructure that research requires.

**Results:**

Existing software solutions provide static and well-established algorithms in a restrictive package. However as high throughput sequencing is a rapidly evolving field, such static approaches lack the ability to readily adopt the latest advances and techniques which are often required by researchers. We have used a loosely coupled, service-oriented infrastructure to develop SeqAdapt. This system streamlines data management and allows for rapid integration of novel algorithms. Our approach also allows computational biologists to focus on developing and applying new methods instead of writing boilerplate infrastructure code.

**Conclusion:**

The system is based around the Addama service architecture and is available at our website as a demonstration web application, an installable single download and as a collection of individual customizable services.

## Background

This paper introduces a flexible and loosely coupled data management system for high throughput sequencing experiments. The system is designed to face the challenges of research, and is required as the versatility and applicability of high throughput sequencing experiments is growing rapidly. The system can be overlaid on top of existing software, and can be used to integrate different specialized algorithms.

There already exist a number of commercial solutions (Geospiza's GeneSifter [1], Genomatix Genome Analyzer [2,3]), and non-commercial solutions (Galaxy [4], CisGenome [5], ChIP-Seq Analysis Server [6]) for the management and analysis of high throughput sequencing information. The main drawback to these solutions is that they focus on providing static "one stop shop" solutions, which are designed to fit known markets, using well-established methods. While these static systems are useful for non-technical researchers in a production science environment, they lack flexibility for the research scientist who wishes to use cutting edge methods and tools.

The existing systems tend to focus on well-established applications for high throughput sequencing: experiments where the technology is seen as a more accurate "digital" equivalent to microarrays (e.g. RNA-Seq), experiments to determine protein binding (e.g. ChIP-Seq), or large scale genome assembly projects. However, high throughput sequencing has the potential of becoming ubiquitous across many avenues of investigation. This potential is due to both an increase in our understanding of systems biology and the capabilities of the new generation of instruments. As the field is constantly evolving new discoveries are continually being made, including new medically related functionality of small RNAs [7], new families of RNA [8], and signaling through extra-cellular RNAs [9]. New techniques and instruments are also being developed that provide insight into these new facets, due to an increase in throughput (e.g. multiplexing [10,11] and long reads [12]) and sophistication (e.g. BS-Seq and targeted approaches). For these reasons, any sequencing software infrastructure used in the research environment must be easily adaptable. By this we mean it must have the ability to be readily changed for new usage. For example, we can expect each research area to require different mechanisms for normalization and replication strategies, sample and experiment vocabularies, and analysis algorithms. Generally within research each project requires a large amount of *de novo *analysis development and customization to support: new technology strategies such as allowing for multiplexing or integrating with new instrumentation; informatics strategies, to allow for data and system integration; and new computational strategies, to support analysis and data-mining tasks. Additionally, each laboratory will have their own demands in terms of experiment QA, annotations and integration with processes (e.g. preferred desktop analysis tools) and integration with other data types. Therefore, it is important that the research community have access to a system that is:

• **Open**. The system must be distributed as an open software project as many users will need to modify the system to meet their specific needs.

• **Standardized**. The system should follow widely used standards for both software development and data exchange. This will ensure that the code base will be easier to maintain and have greater connectivity with external systems and tools.

• **Adaptable**. The system must be easily adaptable without requiring a detailed understanding of the aspects of the internal software architecture. In this way, significant modifications can be implemented efficiently and quickly.

• **Deployable**. The system must be easy to rapidly deploy and modify. A system that is cumbersome or overly complex wastes the end user's development time with unnecessary setup and technical details.

SeqAdapt follows these principles, and provides a standardized and modular architecture which is easy to use, adapt and maintain. The underlying enterprise architecture, Addama [13] has been designed to provide the adaptability required to enable the rapid development needed within research driven science.

## Implementation

To meet the demands of researchers we have developed SeqAdapt, a solution that is able to: scale to meet the requirements of the research environment, use best practices for mainstay applications (e.g. ChIP-Seq), and be readily adapted to new usage.

The system is built using a general software infrastructure to support Adaptable Data Management (Addama). SeqAdapt integrates external sample tracking software (e.g. SLIMseq [14]), workflows for executing analyses (e.g. the MACS algorithm [15]) and robust data management (e.g. JCR) to provide a modular and adaptable system for high throughput sequencing experiments.

**Figure 1:**
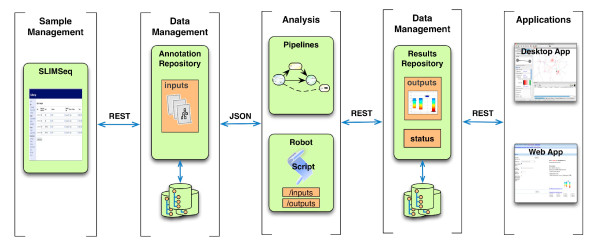
**Architecture of the default SeqAdapt system**. The default SeqAdapt system consist of a number of services and analysis programs that can be replaced to allow for a high level of customization. Default sample tracking, data management, and analysis components are available. These components can be replaced with other systems to suit individual needs. All interfaces between the components are standardized using REST to enable interoperability. The default system uses SLIMseq for initially entering of sample/experiment annotation information, and publishes this information using JSON (over REST). An Addama service accesses the SLIMseq web service and stores the information in a JCR repository. When runs have been completed SLIMseq is updated and this information is pulled into Addama. To run an analysis the Addama Robot system is used, this system allows for any command line utility to be prepared, triggered and monitored. When an analysis is complete the results are automatically pulled into an Addama repository. Customized applications can also be written against the Addama web services.

Due to the data volumes involved with high throughput sequencing a software infrastructure is often required to facilitate storage, management and analysis. We have used the Addama system to provide the necessary support for the creation of a workflow encompassing the entire process (see Figure 1) that is complete, lightweight and easily adapted to changing requirements. This solution allows for changes in the underlying sequencing technology while still providing the ability to plug in new processing methods. A pluggable architecture is important as the technology, data formats, and processing methods are changing rapidly in the field of sequencing. Performance and the ability to scale up as datasets grow in size is also a requirement with the management of sequencing data. To allow for flexible deployment, we use a Service Oriented Architecture (SOA), where distributed services provide the required functions. As these services grow, they can be deployed on the appropriate computational infrastructure (e.g. compute farms, cloud computing environments or dedicated servers).

## Results

The SeqAdapt default download consists of a preconfigured bundle of services. Each of these services can be interchanged with alternative implementations. Additionally the underlying system makes it possible to develop custom applications, and to rapidly integrate new analysis tools (see Integrating New Analyses section).

The software infrastructure is comprised of three main services: a sample tracking system, a data management system and a process management system (see Figure 1). The sample tracking system's functionality includes sample submission, annotation with controlled vocabularies and file management. The data management system uses Addama to organize the data and to trigger new analyses. The process management system (the *Addama Robot*) is a lightweight and generic system for executing processing pipelines and persisting inputs and outputs. The Addama Robot allows for analyses to be run directly on a dedicated machine which has been configured for a specific analysis (e.g. has all the data files, dependencies and resources required for the specific analysis). Typically the analyses are run on either a server machine or, if the analysis is still under in-house development, on the specific developers computer. The robot is responsible for monitoring jobs and the transparent transportation of required data between the repository and the analysis environment.

The data management system uses standard open technologies including: content repositories which are used to generically store all experimental information; information indexing services, which provide for search capabilities across all data and metadata stored; and service registries, which allow for run-time discovery of different content repositories and associated services. Addama also provides an abstraction so that a set of interlinked content repositories can be accessed through a single web application layer. This layer is exposed with a JSON based RESTful web service. The process management system coordinates jobs using a Java Message Service (JMS). With this configuration, the system can scale from a single computer to a distributed set of execution agents on multiple servers to listening to a JMS message queue.

All components of the SeqAdapt system are loosely coupled to allow for easy replacement with alternative systems. These alternative systems could include different sample tracking systems (e.g. Sequencescape [16]) different persistence stores (e.g. RDBMS) and different analysis tools (e.g. RNA-Seq tools).

This system allows for rapid integration of scientific algorithms using the standardized Addama framework. The integration system is designed to be flexible, and allows for any command line analysis tools to be "plugged in". The framework is suited for developing small-scale analyses as well as for large scale processing that requires scaled-up distribution. Further, all components needed for this system are provided in an easy-to-install package.

The default download for SeqAdapt has been set up for Chip-Seq analysis, and will process data from the Illumina Genome Analyzer II using the MACS algorithm (see the Availability section for download details). The default install allows the user to submit an analysis, monitor its progress, and then view the result files.

### Integrating New Analyses

As discussed above, the enterprise system can be extended in a number of ways. As the system is based upon distributed loosely coupled services, these services can be replaced with ones offering the same interface. Each of the major components are built against technology standards (e.g. REST Web Services serve out JSON, the repository is standardized to the JCR specification).

New analysis pipelines can be added by writing a mapping module and then registering the analysis with Addama. A benefit of the Addama systems is that a script can execute in a preexisting development environment, eliminating the time consuming task of replicating software installations on a processing server. When algorithms have reached a mature state and are used widely, the system scales up to have the execution agent installed on many servers. A key benefit of this system is that it manages all of the non-scientific functionality needed in this type of processing. This freedom from writing boilerplate infrastructure code allows the computational biologist to focus on developing the needed scientific software.

**Figure 2:**
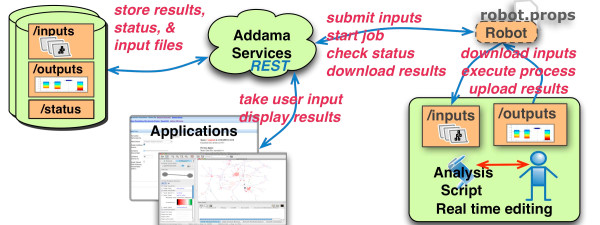
**Integration of analysis tools**. An automated system is used to integrate new analysis tools with Addama. The robot system is responsible for delivering the correct inputs to an analysis script, monitoring the process while it runs, and then for publishing the results of the analysis back. Web applications can then be built on top of standard Addama APIs for providing customizable input information for specific scripts, and then for visualizing the results of the analyses. The loose coupling of each of the web applications from the underlying analysis script makes the system robust to change and (relatively) easy to maintain - especially when the scripts are under constant revision.

The Addama Robot (see Figure 2) allows for the rapid integration of these tools in a relatively short period of time. Integration of these tools requires that the developer has a rudimentary understanding of Addama, and also understands how the specific analysis tool works (in terms of data formats).

By way of example we have integrated the ERANGE [17] RNA-Seq analysis tool. Once ERANGE was installed the integration work was completed in less than a day. Any analysis script that can be run from the command line can be integrated in the same manner. The steps involved in such integration are:

1. ***Define input location. ***This is done by providing a command-line executable wrapper script. This script will define all of the inputs to the ERANGE analysis and execute it. It will read the inputs from a local JSON file downloaded from the Addama service by the Robot.

2. ***Control outputs ***by configuring the wrapper script to write all results to an "/outputs" directory. This directly will be in the same location as the script, the creation of the directory will be handled by the Robot as well. Similarly any log information (e.g. errors, debugging messages) should be written to a "/log" directory.

3. ***Register the script ***by configuring the Addama Robot. The robot uses a properties file that defines the wrapper script that is to be executed, and a local path where each run will be output. Update these properties to reflect the locations of the ERANGE script and the directory where it write inputs, outputs and log messages.

4. ***Enable user submissions***. To make submitting simple for the user, an optional web application may be developed. This application will take the expected inputs and send them to the Addama system via the REST interface. This same page can also be used to query Addama for the results of the Robot analysis and display those for the user as well.

The robot automates the tasks that are required to integrate the analysis with the enterprise system. When the analysis is triggered the robot is responsible for the delivery of the inputs to the analysis, starting the analysis and monitoring the outputs. When completed the outputs, and any associated logs are loaded back, into Addama.

### Walkthrough

**Figure 3:**
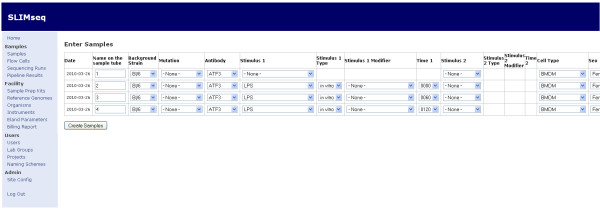
**Step 1: Sample Entry**. Sample information is entered into the custom ordering system SLIMseq.

**Figure 4:**
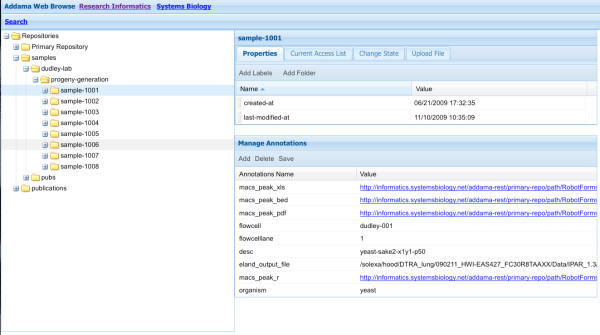
**Step 2: Browsing**. A repository stores all the annotations and sample data files so they can be searched and browsed.

**Figure 5:**
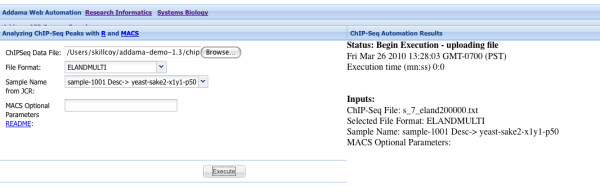
**Step 3: Analysis**. Data stored within the repository can be analyzed with any integrated analysis. In this case MACS can be triggered and the results automatically stored back in Addama.

**Figure 6:**
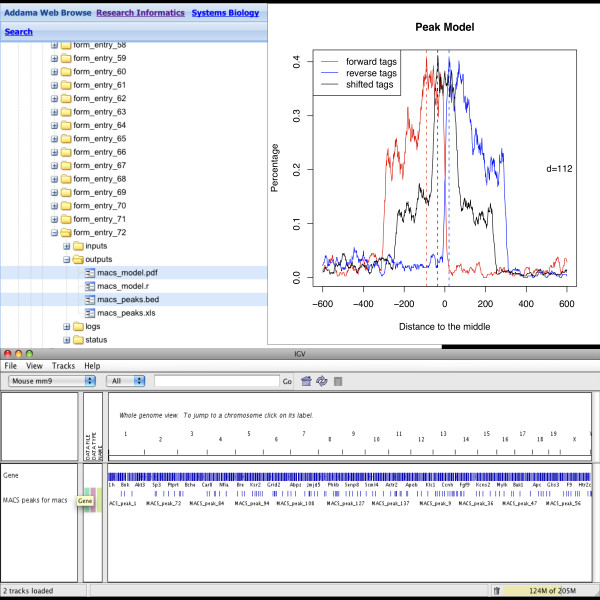
**Step 4: Browsing**. The results of an analysis are stored in Addama and can be integrated with other data files and viewed in applications such as IGV.

A walk-through showing the default workflow for SeqAdapt is given in Figures 3,4,5 and 6. This walk-through shows how the system can be used to capture information about a Chip-Seq experiment, store the results and then analyze the reads using MACS.

## Conclusions

The default system can be used "as-is" to support ChIP-Seq analysis, however it can also be rapidly customized to suit new usage. The requirement for such flexibility is due to the fact that it is rarely possible to foresee all the usages with which a new instrument technology, such as high throughput sequencing, will be applied.

To be able to meet the requirements of a rapidly evolving research environment, as is found in most scientific institutions, an adaptable data management system is required. The modular framework described in this article is designed to provide such adaptability. The major advantage of the SeqAdapt/Addama system is that "ad hoc" tools can be rapidly integrated. In this instance "ad hoc" means tools that do not adhere to predefined standards and have been built without integration in mind (e.g. not provided as web services).

The system is available from the Institute for Systems Biology's informatics web site, as: a *demonstration *system running as a web application, a *preconfigured install *for quick deployment and as a *full download *containing all the separate services (see Availability, below). If access control is a requirement then the full download should be used.

## Availability and Requirements

Project name: SeqAdapt.

Project home page: http://informatics.systemsbiology.net/informatics/seqadapt

Operating system(s): Platform independent.

Programming language: Java; Other requirements: Java 1.6 or higher.

Licence: Apache 2.0.

Any restrictions to use by non-academics: None.

Full installation instructions and software downloads are available at the project web site.

## Abbreviations

**JSON (JavaScript Object Notation)**: A simple object serialization format originally designed to work with JavaScript. JSON is growing in popularity over other text based formats (e.g. XML) due to its lightweight and simplistic nature. JSON encoders and parsers are available in most languages; **SOA (Service Oriented Architecture)**: A software architecture method where modules of software are separated into services that expose an interface of functionality. These modules can be combined in several ways to create larger distributed applications; **Web Service**: A software system that supports interaction over a network. Web services are typically exposed using HTTP; **JMS (Java Message Service)**: A Java API standard that allows Java applications to exchange messages. It allows for distributed communication that is loosely coupled and asynchronous; **REST (REpresentational State Transfer)**: An architecture for distributed content, such as the World Wide Web. A RESTful web service uses standard HTTP constructs (e.g. GET, POST, DELETE) to provide services, and communicates back to the client using HTTP response codes (e.g. 200 - OK, 404 - Not Found) along with content; **JCR (Java Content Repository)**: A type of object store that is suited towards the storage, searching and retrieval of hierarchical data. A JCR can store both metadata and as well as files;

## Authors' contributions

SR and DB developed the initial ChIPSeq analysis, and translated that work into a pipeline. BM developed the SLIMseq suite and consulted on pipeline development, HR, DB, CC, SK, JH and JL developed the Addama software. RB and JL provided the installation package, SK, JH and JL tested and wrote the installation and use instructions. IS and JB conceived of the study, and participated in its design and coordination. All authors read and approved the final manuscript.

## References

[B1] Geospiza http://www.genesifter.net/

[B2] Werner T Krawetz S The Role of Transcription Factor Binding Sites in Promoters and Their In Silico Detection Bioinformatics for Systems Biology 2009 339 352 full_text

[B3] Genomatix Genome Analyzer http://www.genomatix.de/en/produkte/genomatix-genome-analyzer.html

[B4] Taylor J Using galaxy to perform large-scale interactive data analyses Curr Protoc Bioinformatics 2007 10 18428782 10.1002/0471250953.bi1005s19 PMC3418382

[B5] Ji H An integrated software system for analyzing ChIP-chip and ChIP-seq data Nat Biotechnol 2008 26 11 1293 1300 10.1038/nbt.1505 18978777 PMC2596672

[B6] ChIP-Seq Analysis Server http://www.isrec.isb-sib.ch/chipseq/

[B7] Grosshans H Filipowicz W Molecular biology: the expanding world of small RNAs Nature 2008 451 414 416 10.1038/451414a 18216846

[B8] Taft RJ Tiny RNAs associated with transcription start sites in animals Nat Genet 2009 41 5 572 8 10.1038/ng.312 19377478

[B9] Dinger ME Mercer TR Mattick JS RNAs as extracellular signaling molecules J Mol Endocrinol 2008 40 4 151 9 10.1677/JME-07-0160 18372404

[B10] Cronn R Multiplex sequencing of plant chloroplast genomes using Solexa sequencing-by-synthesis technology Nucleic Acids Res 2008 36 19 e122 10.1093/nar/gkn502 18753151 PMC2577356

[B11] Parameswaran P A pyrosequencing-tailored nucleotide barcode design unveils opportunities for large-scale sample multiplexing Nucleic Acids Res 2007 35 19 e130 10.1093/nar/gkm760 17932070 PMC2095802

[B12] Eid J Real-time DNA sequencing from single polymerase molecules Science 2009 323 5910 133 8 10.1126/science.1162986 19023044

[B13] Boyle J Adaptable Data Management for Systems Biology Investigations BMC Bioinformatics 2009 10 79 19265554 10.1186/1471-2105-10-79 PMC2670281

[B14] Marzolf B Troisch P SLIMarray: Lightweight software for microarray facility management Source Code for Biology and Medicine 2006 1 5 17147785 10.1186/1751-0473-1-5 PMC1636632

[B15] Zhang Y Model-based analysis of ChIP-Seq (MACS) Genome Biol 2008 9 9 R137 10.1186/gb-2008-9-9-r137 18798982 PMC2592715

[B16] Jorgensen L Production Software Development at Wellcome Trust Sanger Institute (Sequencsescape) Data Integration in the Life Sciences Workshops (DILS) 2009 18

[B17] Mortazavi A Mapping and quantifying mammalian transcriptomes by RNA-Seq Nat Methods 2008 5 7 621 8 10.1038/nmeth.1226 18516045 PMC13303166

